# P2X7 mRNA expression in non-small cell lung cancer: MicroRNA regulation and prognostic value

**DOI:** 10.3892/ol.2014.2620

**Published:** 2014-10-15

**Authors:** LAURA BOLDRINI, MIRELLA GIORDANO, GRETA ALÌ, FRANCA MELFI, GAETANO ROMANO, MARCO LUCCHI, GABRIELLA FONTANINI

**Affiliations:** 1 Department of Surgical, Medical, Molecular Pathology and Critical Area, University of Pisa, Pisa 56126, Italy; 2 Unit of Pathological Anatomy III, University Hospital of Pisa, Pisa 56126, Italy; 3 Unit of Thoracic Surgery, University Hospital of Pisa, Pisa 56126, Italy

**Keywords:** P2X7, miRNA, non-small cell lung cancer, prognosis

## Abstract

The human P2X7 receptor is significant and exhibits several functions in neoplasia. At present, little is known with regard to its regulation. P2X7 expression may be regulated post-transcriptionally and putative microRNA (miRNA) binding sites are considered to be involved. The aim of this study was to determine whether miRNAs (miR-21, let-7 g and miR-205) regulate P2X7 mRNA stability. In addition, the impact of P2X7 expression in patients with non-small cell lung cancer (NSCLC) was investigated. P2X7 mRNA and mature Let-7 g, miR-21, and miR-205 expression levels were quantified in 96 NSCLC cases using quantitative reverse transcription polymerase chain reaction. In all samples, epidermal growth factor receptor and K-Ras mutational analysis was also performed. Samples with low P2X7 expression were found to exhibit a higher fold change in miR-21 expression when compared with samples exhibiting high P2X7 expression. Significantly higher miR-21 expression was observed in the tumors of NSCLC patients with a K-Ras mutation when compared with patients who had K-Ras wild-type tumors (P=0.003). Additionally, to evaluate the association between P2X7 expression and prognosis in NSCLC patients, survival analysis was performed using the Kaplan-Meier method. A significant difference in the progression-free survival and overall survival in the NSCLC patients with high P2X7 expression was identified, when compared with that of patients with low expression (P=0.03 and P=0.02, respetively). Therefore, we hypothesized that high levels of miR-21 expression in NSCLC patients with K-Ras mutations may be regulated by a complex circuit, including P2X7 downregulation and together these processes may promote tumor progression.

## Introduction

The human P2X7 receptor is a trimeric ligand-gated cation channel encoded by the P2RX7 gene, which is located on chromosome 12q24 (1). P2X7 is expressed in a wide variety of normal and disease-associated cell types. Activation of this receptor by extracellular adenosine 5′-triphosphate results in numerous downstream events, including the release of proinflammatory mediators, cell proliferation or death, and killing of intracellular pathogens. As a result, P2X7 is significant and exhibits several functions in neoplasia. P2X7 and its regulation is of considerable interest with regard to human health and disease, including in the development and publication of a number of patents (2). At present, little is known with regard to how epithelial cells regulate expression of the P2X7 receptor. However, a preliminary study revealed that P2X7 expression is also regulated post-transcriptionally (3). In addition, putative microRNA (miRNA) binding sites within the P2RX7 3′-untranslated region (UTR) have also been identified (4,5). miRNAs are small, noncoding 18–25-nucleotide RNAs that regulate mRNA targets and control critical functions in a variety of biological processes (6). Animal miRNAs form imperfectly base-paired duplexes with miRNA response elements in target mRNAs. This interaction may inhibit translation and lead to degradation of mRNA (7,8). For effective repression, base-pairing between the miRNA-response elements and the first 2–7 nucleotides of the miRNA, referred to as the ‘seed’ region, is important (9–11). miR-21 is overexpressed in numerous types of tumors, including non-small cell lung cancer (NSCLC), indicating its significance in cancer development. However, the underlying mechanism of miR-21-mediated tumorigenesis remains unclear, largely due to limited knowledge with regard to miR-21 targets. Various computer-aided algorithms have predicted a number of putative miR-21 targets, however, these targets have not been validated experimentally. The aim of this study was to investigate whether miRNAs (miR-21, let-7 g, and miR-205) regulate P2X7 mRNA stability and to evaluate the prognostic impact of P2RX7 expression in patients with NSCLC.

## Materials and methods

### Patients

In total, 96 NSCLC patients were retrospectively selected from patients who had undergone resection at the Unit of Thoracic Surgery of Pisa, Azienda Ospedaliero-Universitaria Pisana (Pisa, Italy) between 1993 and 2012. Histological diagnoses were formulated according to the World Health Organization classification (12,13). Clinicopathological characteristics were collected in 82 cases, while survival data were obtained for 53 patients. All patients provided written informed consent prior to the molecular analyses. This study was approved by the ethics committee of the University of Pisa (Pisa, Italy).

### RNA and DNA isolation

RNA and DNA were isolated from 5–10 μm sections of 82 formalin-fixed paraffin-embedded (FFPE) tissues and 14 cytological specimens. The cytological samples were obtained from bronchial brushing, aspirative transbronchial needle aspiration and transthoracic needle aspiration. The cyctolgical preparations were conducted according to a standard specimen processing procedure in our laboratory. All samples were fixed in Cytofix (BD Biosciences, San Jose, CA, USA) and stained with Papanicolaou for 20 min in an automated stainer (Leica Stainer; ST5020, Leica, Mannheim, Germany). For aspirative cytology (transbronchial needle aspiration and transthoracic needle aspiration), the specimens were fixed in Cytofix and stained with Papanicolaou staining (Kaltek s.r.l., Padua, Italy). Bronchial brushing samples and cellular material were centrifuged (Sorvall T6000D, Thermo Scientific, Rockford, IL, USA) at 1366 x g for 10 min. Next, 10% neutral buffered formalin (Diapath S.p.A., Bergamo, Italy) was added to the pellet and the pellet placed into a tissue processing and embedding cassette (Bio Optica S.p.A., Milan, Italy) and embedded with paraffin.

### P2X7, miRNA expression and mutational analysis

Quantification of mRNA expression was performed in triplicate using quantitative reverse transcription polymerase chain reaction (PCR) with the following primers: Forward, 5′-CTCCCATCTCAACTCCCTGA-3′ and reverse, 5′ACCAGCTTCCTGAACAGCTC-3′, for P2X7. Specific TaqMan^®^ miRNA Assays (Applied Biosystems Life Technologies, Foster City, CA, USA) were used for Let-7 g, miR-21, miR-205 and RNU6B according to the manufacturer’s instructions. The threshold cycle (Ct) and baselines were determined using manual settings, where Ct was set in the linear phase of the amplification and baseline in the initial cycles of PCR. Expression was calculated by relative quantification and fold expression changes were determined by the 2^−ΔΔ^*^C^*^T^ method using the DataAssist software (Applied Biosystems Life Technologies).

Mutational analysis was also performed for all samples. The mutational status of the codons 12–13 of the K-Ras gene was analyzed by pyrosequencing using the anti-epidermal growth factor receptor (EGFR) MoAb response^®^ kit (K-Ras status) (Diatech Pharmacogenetics, Jesi, Italy) according to the manufacturer’s instructions.

PCR-single stranded conformation polymorphism and sequencing analysis were used for EGFR genotyping in exons 18–21, as previously described (14).

### Statistical analysis

One-way analysis of variance and χ^2^ tests were used to determine the association between miRNA expression, P2X7 mRNA expression and the different parameters. The survival analysis was performed using the Kaplan-Meier method. Statistical analyses were performed using the JMP10 software (SAS, Cary, NC, USA) and a two-tailed P<0.05 was considered to indicate a statistically significant difference.

## Results

### Patient characteristics

This study was conducted in 96 patients with NSCLC, which included 63 adenocarcinomas, 31 squamous cell carcinomas and two large cell carcinomas. The median age at diagnosis was 66.5 years (range, 46–83 years). Using the TNM staging system, the distribution of the patients was as follows: 18 cases, stage I; 44 cases, stage II; 14 cases, stage III; and six cases, stage IV; 34 cases, negative lymph node status; 20 cases, N1 status; 23 cases, N2 status; and five cases, Nx status (13).

Survival data were obtained for 53 patients (median follow-up duration, 36 months; range, 7–98 months). Disease progression and mortality from lung cancer were observed in 32 (60.3%) and 18 (34%) of the 53 NSCLC patients, respectively. The median progression-free survival (PFS) and overall survival (OS) were 18 months (95% CI, 2–89 months) and 25 months (95% CI, 2–90 months), respectively.

### P2X7 and miRNA expression and clinicopathological characteristics

P2X7 mRNA expression was quantified and normalized to the glyceraldehyde 3-phosphate-dehydrogenase housekeeping gene. The expression of mature Let-7 g, miR-21, and miR-205 was quantified and normalized to the RNU6B endogenous control in 96 NSCLC tissues using quantitative PCR. The samples were divided into high and low expression groups based on the median fold change values (0.56, P2X7; 1.02, Let-7 g; 5.81, miR-21; and 0.01, miR-205). No statistically significant associations were identified between P2X7 mRNA levels and the main clinicopathological characteristics of the NSCLC patients (Table I). With regard to the association between P2X7 expression levels and the miRNA profile, it was found that samples with low P2X7 expression exhibited a higher miR-21 fold change (11.42±2.28) when compared with samples expressing high levels of P2X7 (7.41±2.28). In addition, the mutational analysis revealed K-Ras and EGFR mutations in 19.7 and 23.9% of the NSCLC patients, respectively. K-Ras and EGFR mutations were mutually exclusive (P=0.0006), observed only in the NSCLC patients with adenocarcinoma (P<0.0001). The mutations were associated with female gender (P<0.0001) and patients that did not smoke (P<0.0001). Notably, significantly higher miR-21 expression was observed in the NSCLC tumors that expressed mutant K-Ras when compared to that of tumors that expressed wild-type K-Ras (P=0.003). In addition, the majority of the tumors that expressed mutant K-Ras exhibited low P2X7 expression.

### Survival analysis

To evaluate the association between P2X7 expression and the prognosis of NSCLC patients, a survival analysis using the Kaplan-Meier method was conducted. In this analysis, disease recurrence and overall postoperative survival were used as endpoints.

**Figure 1:**
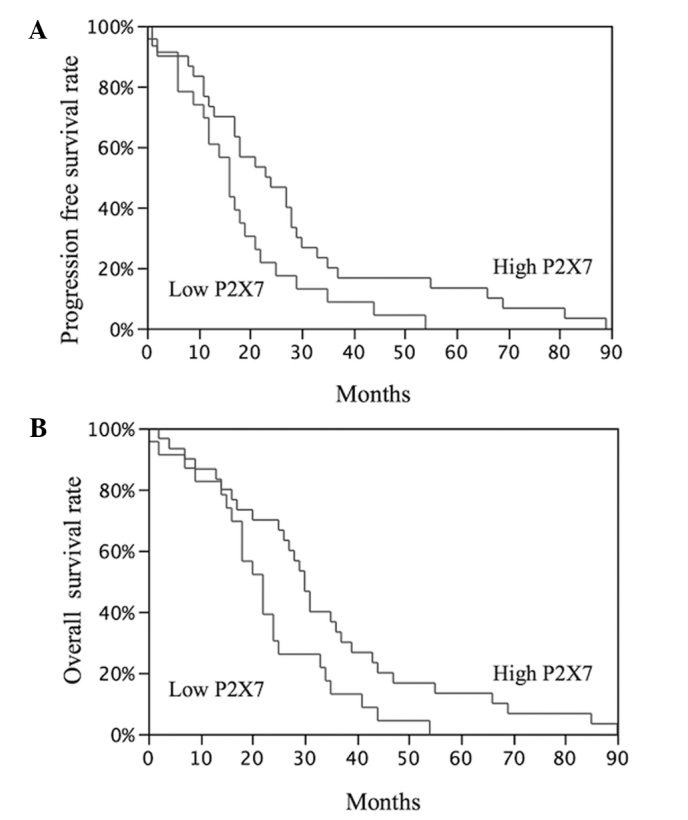
Kaplan-Meier curves for survival analysis of (A) PFS and (B) OS in non-small cell lung cancer patients with different P2X7 mRNA expression levels. P=0.03, log-Rank test for PFS; P=0.02, log-Rank test for OS. PFS, progression free survival; OS, overall survival.

A significant difference was identified between the PFS (P=0.03) and OS (P=0.02) of the NSCLC patients with high P2X7 expression and the patients with low P2X7 expression (Fig. 1).

## Discussion

P2X7 is characterized by high plasticity, as its expression is modulated in a cell type- or differentiation-dependent manner (15). Transcriptional mechanisms may underlie the increased P2X7 expression observed in lung cancer, and the expression of individual isoforms of the same protein may or may not correlate with the mRNA, indicating that separate post-translational mechanisms may account for the regulation of P2X7 expression (2). In the current study, P2X7 mRNA levels were evaluated and quantified by quantitative PCR in NSCLC cases. One of the aims of this study was to explore whether particular miRNAs (miR-21, let-7 g and miR-205) are important for P2X7 mRNA regulation. Samples with low P2X7 expression were found to exhibit a higher miR-21 fold change when compared with samples exhibiting high levels of P2X7 expression. In animals, miRNAs are hypothesized to bind via a partially homologous sequence in the target gene at the 3′-UTR, causing translational repression (6). With regard to oncogenic miRNAs, a limited number of target genes have been characterized experimentally, however, increasing evidence indicates putative targets, predicted by different algorithm programs. For example, the Sanger miRNA database target search (www.mirbase.org.) reveals >900 targets for miR-21, which is not consistent with the prediction of ~100 target genes per single miRNA (16). Therefore, it is likely that only a small fraction of predicted targets may be true targets and thus, it would be difficult to validate them. In the present study, an exact putative target site was not identified for miR-21 within the 3′-UTR (1272 nucleotides) of the human P2X7 gene (NM_002562). However, at the 499 nucleotide position, a partial target site (3′-GAAT-5′) was identified for the miR-21 seed sequence (5′-AGCUUA-3′). Further studies are required to investigate whether P2X7 is a target of miR-21, considering that in animals, the miRNA does not bind to its target as efficiently as in plants. Furthermore, polymorphisms in the miRNA ‘seed region’ (5) or in their binding sites in target genes have been identified and appear to be involved in cancer (17).

Furthermore, significantly higher miR-21 expression was observed in NSCLC patients with K-Ras-mutated tumors, when compared with K-Ras wild-type specimens, according to our previous study (18). High levels of miR-21 expression in NSCLC patients harboring K-Ras mutations may indicate a synergistic assocation between miR-21 and K-Ras oncogenes, including P2X7 downregulation. Taken together, it is likely that these processes promote tumor progression. The second aim of the study was to evaluate the putative prognostic role for P2X7 expression, as this role remains controversial. Based on previous studies, it appears that low levels of P2X7 activation facilitates the growth or survival of certain tumor cell types (19). However, at high levels, the extracellular receptor promotes cell death (20). Previous reports have linked increased P2X7 expression with a poor prognosis in several types of leukemia (21,22) and solid tumors (23–25). By contrast, Souza *et al* (26) revealed that extracellular ATP effectively inhibited proliferation and induced apoptosis or necrosis of tumor cells (26). These studies also demonstrated that the brief exposure of tumor cells to ATP was able to efficiently induce cell death (reduction of cell growth and induction of autophagy), which was largely mediated via P2X7, indicating the anti-tumor potential of purine-based drugs (27). The results of the current study are consistent with those found by Souza *et al* (26), showing that defective P2X7 expression, as a result of miR-21 activation by a K-Ras mutation, may lead to reduced tumor-killing activity, resulting in a poorer prognosis. The identification of putative associations of P2X7 with biological behavior in NSCLC would be of considerable interest, and further studies will aid in the understanding of P2X7 gene regulation and its role in lung cancer. The significant differences in clinical outcome of NSCLC patients with high P2X7 expression identified in this study indicate that expression of the P2X7 receptor may be a useful prognostic marker, as well as a novel target for therapy. Further studies, including the investigation of P2X7 regulation by various micro-RNA or other epigenetic mechanisms, may provide more insight with regard to the results of this study.

## Figures and Tables

**Table I:** Correlations between P2X7 mRNA expression and the main clinicopathological characteristics of the non-small cell lung cancer patients.

	P2X7 mRNA expression, n (%)		
		
Parameter	Low	High	P-value
Age, years			
≤68	28 (56)	22 (44)	0.21
>68	20 (43.5)	26 (56.5)	
Gender, n			
Male	33 (45.8)	39 (54.2)	0.15
Female	15 (62.5)	9 (37.5)	
Histology, n			
ADC	32 (50.8)	31 (49.2)	0.21
SCC	14 (45.2)	17 (54.8)	
LCC	2 (100)	0 (0)	
Tumor stage^a^, n			
T1 (T1a–T1b)	6 (46.1)	7 (53.9)	0.70
T2 (T2a–T2b)	25 (51)	24 (49)	
T3–T4	8 (40)	12 (60)	
Lymph-node status, n			
Negative	21 (61.8)	13 (38.2)	0.09
Positive	16 (37.2)	27 (62.8)	
Nx	2 (40)	3 (60)	

a TNM staging system.

ADC, adenocarcinoma; SCC, squamous cell carcinoma; LCC, large cell carcinoma.
